# Development and validation of a novel immune-related prognostic model and the potential metastatic mechanism in synovial sarcoma

**DOI:** 10.3389/fimmu.2024.1448464

**Published:** 2024-12-10

**Authors:** Yufeng Huang, Ming Gong, Jiaming Lin, Qinglian Tang, Hongmin Chen, Jinxin Hu, Chuangzhong Deng, Anfei Huang, Huixiong Feng, Guohui Song, Huaiyuan Xu, Jinchang Lu, Xiaojun Zhu, Jin Wang

**Affiliations:** ^1^ Department of Cervical Spondylosis and Spine Orthopedics, The First Affiliated Hospital of Guangzhou University of Chinese Medicine, Guangzhou, China; ^2^ Guangdong Clinical Research Academy of Chinese Medicine, Guangzhou, China; ^3^ State Key Laboratory of Oncology in Southern China, Collaborative Innovation Center of Cancer Medicine, Guangzhou, China; ^4^ Department of Pediatric Orthopaedics, GuangZhou Women and Children’s Medical Center, GuangZhou Medical University, Guangdong Provincial Clinical Research Center for Child Health, Guangzhou, China; ^5^ Department of Musculoskeletal Oncology, Sun Yat-sen University Cancer Center, Guangzhou, China

**Keywords:** synovial sarcoma (SS), immune-related genes, immune checkpoints (ICPs), prognosis, tumor microenvironment (TME)

## Abstract

**Background:**

Several clinical trials have shown that immunotherapy plays a pivotal role in the treatment of patients with metastatic synovial sarcoma. Immune-related genes (IRGs) have been demonstrated to predict the immunotherapy response in certain malignant tumours. However, the clinical significance of IRGs in patients with synovial sarcoma (SS) is still unclear.

**Methods:**

We first combined the immune-related ImmPort gene set to search for SS related to metastatic and differentially expressed immune-related genes (DEIRGs) in the GSE40021 dataset from the GEO database. The soft tissue sarcoma database in TCGA was used for univariate Cox regression analyses to identify DEIRGs that were related to overall survival and to build an immune-related prognostic assessment model.

**Results:**

The study screened a total of six DEIRGs that were closely related to prognosis in metastatic SS. Further analysis showed that there was no significant difference in the expression of several immune checkpoints between the two groups in the GSE40021 data. Moreover, the GREM2 and CTSS genes were significantly expressed in metastatic patients. Further verification of clinical SS tissues from our centre by RT-qPCR analysis demonstrated reduced infiltration of activated NK cells and macrophages but increased M2-type macrophages in metastatic patients. Together, our study successfully constructed an immune-related prognostic assessment model and probably explains the poor efficacy of PD-1 inhibitors for SS patients.

**Conclusion:**

The research deepens our understanding of the tumor immune microenvironment and proposes a new immune mechanism for metastatic SS. Early intervention and reversal of immunosuppressive microenvironmental changes are expected to delay metastasis and improve survival.

## 1 Introduction

Synovial sarcoma(SS) is a rare and aggressive malignant tumor derived from spindle-shaped mesenchymal tissue. The specific origin of this tumor tissue type has not been confirmed, but current research suggests that it may be derived from myoblasts and nerve or primitive mesenchymal cells (1). Genetically, synovial sarcoma has a specific t(X;18)(p11.2;q11.2) gene translocation and produces the SS18-SSX fusion gene. Synovial sarcoma accounts for 5% to 10% of soft tissue sarcomas, and the age of onset is mainly 15-40 years old (2, 3). SS is the most common soft tissue sarcoma in young people, except rhabdomyosarcoma. The current treatment of synovial sarcoma involves neoadjuvant chemotherapy combined with extensive surgical resection, and adjuvant chemotherapy or radiotherapy is also utilized according to the tumor stage and prognostic factors (4).

SS and other soft tissue sarcomas have different biological behaviors, prognoses, and treatments. For example, SS is more sensitive to chemotherapy, and the molecular and immunotherapy targets are different. The lung is the most common site of initial recurrence after treatment. Once metastasis occurs, the median survival time is approximately 1 year (5). For patients with metastases who are resistant to advanced chemotherapy, immunotherapy may be one of the possible effective treatments. In the SARC028 clinical trial, patients with metastatic or surgically unresectable locally advanced sarcoma were treated with pembrolizumab(200mg) intravenously every three weeks, and the main endpoint of the study was the objective response rate of the tumor to the treatment. The results showed that undifferentiated sarcoma (UPS), myxoid liposarcoma(LPS), and SS had ORRs of 40%, 20%, and 10%,respectively (6). In the clinical trial of ALLIANCE A091401, the patients with advanced soft tissue sarcoma were included in the same group to evaluate the objective response rate of nivolumab alone or in combination with ipilimumab. Among the effective types of sarcoma, the single-agent treatment group included Alveolar soft part sarcoma(ASPS) and Leiomyosarcoma(LMS), and the combined treatment group included undifferentiated sarcoma(UPS), Leiomyosarcoma(LMS), myxofibrosarcoma(MFS), and Anigosarcoma(AS) (7). However, two subsequent clinical trials with expanded samples did not include SS. In adoptive cellular immunotherapy, up to 80% of patients with SS express NY-ESO-1 antigen, while the expression level is low in other normal mesenchymal cells. D’Angelo and colleagues genetically engineered autologous T cell receptors to express NY-ESO-1 antibody to enhance the recognition and killing of target antigens. This clinical trial showed good safety and effectiveness, of which 50% (6/12) of patients showed an antitumor response (8). Immunotherapy plays a pivotal role in patients with advanced synovial sarcoma, but screening these patients with effective immunotherapy has become a key issue in clinical treatment decision-making. At present, there is no definite biomarker or model that is effective in predicting the prognosis of advanced SS.

Based on bioinformatics analysis, the study aimed to screen the key metastatic genes associated with immune-related SS and to construct an immune-related prognostic model. First, we used the GSE40021 dataset combined with the ImmPort gene set to screen the DEIRGs of metastatic SS and performed functional enrichment analysis. We then used the soft tissue sarcoma database in TCGA to further screen the genes that were significantly related to prognosis to construct a risk assessment model. We also utilized the ssGSEA algorithm to compare immune cell infiltration in the tumor microenvironment between patients with high-risk scores and metastasis. We found that the infiltration of immune cell subpopulations overlapped significantly between the two groups. Among them, eosinophils, CD4+T lymphocytes, NK cells, and macrophages were significantly infiltrated in SS with poor prognosis, while the expression of most immune checkpoints were not significantly different. Finally, we found that the GREM2 and CTSS expressions(the other four genes were not significantly different) were significantly increased in metastatic SS from our center. In addition, the infiltration of activated NK cells was reduced, but M2 macrophages were increased in these patients.

The study successfully constructed an immune-related prognostic assessment model for metastatic SS that can predict the prognosis of patients and screen patients who may benefit from immunotherapy. By analyzing the infiltration of immune cells in the tumor microenvironment, we found that the immune metastatic mechanism may be associated with the polarization of M2 macrophages and the remodeling of the immune microenvironment. This process leads to a decrease in NK cell infiltration without promoting the escape of immune checkpoints. The model offers significant anti-metastatic therapeutic options for patients with advanced SS, potentially reducing medical costs and delaying metastasis to distant organs, thereby improving overall patient survival. Consequently, the model holds considerable significance for clinical treatment decisions.

## 2 Materials and methods

### 2.1 Patients and datasets

Clinical data: Patients with synovial sarcoma who were admitted to the Department of Bone and Soft Tissue in our hospital from May 2017 to May 2018 and had at least three years of follow-up data were enrolled. According to statistics, there were 11 nonmetastatic cases and 5 metastatic cases. The general clinical data of the patients are shown in **Table 1**. Clinical data of high-level soft tissue sarcoma patients were downloaded from TCGA database. General clinical information was obtained for 259 patients (**Table 2**).

**Table 1:** The general clinical data of the SS patients.

Non-metastasis						
Patient		Sex	Age	Location	Date of surgery	Chemotherapy response
1		M	35	Right leg	2017/05/21	PR
2		F	26	Left leg	2017/06/01	PR
3		M	38	Left waist	2017/08/07	PR
4		F	48	Right arm	2017/09/21	PD
5		M	19	Right popliteal fossa	2018/01/23	PD
6		M	53	Right leg	2018/01/28	PD
7		M	36	Right leg	2018/02/04	SD
8		F	36	Right abdominal wall	2018/03/07	PR
9		M	35	Left leg	2018/03/25	PR
10		F	34	Left armpit	2018/04/07	PR
11		F	16	Left groin	2018/04/20	SD
Metastasis						
Name	Sex	Age	Location	Date of surgery	Chemotherapy response	Metastasis site
1	M	53	Pelvis	2017/11/11	PD	Lung
2	F	18	Right groin	2017/12/04	PR	Lung
3	M	12	Left political Formosa	2018/01/20	PD	Lung and Right inguinal lymph node
4	F	48	Right leg	2018/03/21	PR	Lung
5	F	14	Left shoulder	2018/05/08	PD	Lung

**Table 2:** Clinical data of high-level soft tissue sarcoma patients.

	Total set (n=259)	Training set (n=181)	Validation set (n=78)
Age, years	60.56	60.38	61
Race			
White	226	157	69
Other	6	2	4
Unknown	27	22	5
Sex			
Male	118	78	40
Female	141	103	38
Tumor site			
Extremity	85	60	25
Other	174	121	53
Margin status			
R0	154	107	47
R1/2	78	55	23
Unknown	27	19	8
Metastasis			
NO	120	77	43
YES	56	40	16
Unknown	83	64	19
Radiotherapy			
NO	140	90	50
YES	73	56	17
Unknown	46	35	11
Histological			
LMS	104	81	23
DLPS	58	36	22
UPS	51	33	18
MFS	25	17	8
SS	10	8	2
Other	11	6	5

LMS, Leiomyosarcoma; DLPS, Dedifferentiated liposarcoma.

UPS, undifferentiated pleomorphic sarcoma.

MFS, Myxofibrosarcoma; SS, Synovial sarcoma.

RNA-seq data: The expression profile data for the GSE40021 dataset were obtained from the GEO database (https://www.ncbi.nlm.nih.gov/geo/), which is based on the GPL6480 platform (Agilent-014850 Whole Human Genome Microarray 4× 44K G4112F), including 28 cases of synovial sarcoma tissue samples that had metastasized at the time of consultation and 30 cases of non-metastatic samples.

### 2.2 Identification of differentially expressed immune-related genes and enrichment analysis

To screen for metastatic genes associated with immune-related synovial sarcoma, we used the GEO2R online web tool, which allows users to compare the expression data of different genes between metastatic and non-metastatic samples. We used the Wilcoxon test method and set the corrected P value<0.05 and |log2-fold change|≥1.0 as significant for the DEGs. Among them, DEGs with logFC>0 were considered to be upregulated, while DEGs with logFC<0 were considered to be downregulated. We also download 1811 immune-related genes via the Immunology Database and Analysis Portal (ImmPort; https://www.immport.org/shared/genelists) database, which contains 17 immune categories based on various molecular function (9). Finally, we took the intersection between DEGs and Immune-related gene sets (IRGs) in ImmPort gene sets to obtain DEIRGs. Heatmaps were generated using pheatmap package and volcano plots were also conducted in R software.

To evaluate the potential biologic functions of DEIRGs, Gene Ontology (GO) (10) and Kyoto Encyclopedia of Genes and Genomes (KEGG) pathway enrichment analysis (11) were performed by the cluster Profiler package in R. Functional categories with a adjusted P value<0.05 were considered as significant pathways. GO function enrichment included biological process (BP), cell component (CC), and molecular function (MF). The top 10 functional enrichment results and signalling pathways were selected.

The DEIRGs list was used for protein–protein interaction (PPI) analysis in Cytoscape (v3.7.1, National Resource for Network Biology, https://cytoscape.org/) with default parameters (12). Only individual networks with more than 10 nodes were included for Molecular COmplex DEtection (MCODE).

### 2.3 Survival analysis

To investigate the prognostic value of DEIRGs in SS patients, high-grade soft tissue sarcoma data in TCGA database were used to further screen the DEIRGs related to prognosis. Univariate Cox analysis was implemented by the survival package. Only these genes with a P value < 0.01 were considered as prognostic immune-related genes.

### 2.4 Construction of the immune-related prognostic signature for SS

Due to the limited availability of genomic studies involving large samples of synovial sarcoma patients in current open-source databases, we believe that the high-grade sarcoma database from the Cancer Genome Atlas (TCGA) can partially represent the characteristics of SS patients. Consequently, we selected the TCGA-SARC dataset for subsequent model validation analysis. However, following a thorough pathological verification of the original TCGA-SARC dataset, we discovered that some samples had been misdiagnosed (13). Therefore, we quantified the proportion of erroneous cases in both the validation and training sets within their respective datasets and conducted a comparative analysis. The results indicated no significant difference between the two groups, suggesting that erroneous cases do not hinder the model’s capacity to evaluate patient prognosis in the TCGA-SARC original dataset (**Table 3**). Overall, the method employed to validate predictive models using this dataset appears to be viable. Previous literature has similarly reported that a comprehensive analysis of the immune regulatory gene landscape utilizing the TCGA-SARC original data has identified novel immune regulatory genes (IRGs) associated with carcinogenesis and the immune microenvironment (38). It is plausible that this literature also employed the aforementioned statistical analysis method to address data-related challenges.

**Table 3:** Comparative analysis of the proportion of misdiagnosed cases.

TCGA-sarc	Correct cases	Incorrect cases	Total	Proportion of incorrect cases	P-value
Training cohort	150	31	181	17.1%	0.0638 (ns) (Fisher,s exact test)
Validation cohort	56	22	78	28.2%	
Total	206	53	259		

To develop a prognostic model, Lasso and multivariate Cox regression analyses were utilized to assess the relationship between DEIRGs expressions and overall survival (OS) or metastasis-free survival(MFS). $risk\_score=\sum_{i=1}^{n}coef_{i}*X_{i}$ . Coef refers to regression coefficient and n is the number of prognostic-related DEIRGs. According to the formula, the risk score of each patient was calculated. Risk scores were acquired based on genes expression multiplied a linear combination of regression coefficient obtained from the multivariate Cox regression. Patients were assigned to high risk and low risk groups according to the median risk score. The Kaplan-Meier analysis was performed to compare overall survival between high risk and low risk groups via survival package in R. Using the high grade soft tissue sarcoma dataset in TCGA, the receiver operating characteristic (ROC) curve was performed by the R software package survival ROC. In addition, univariate and multivariate analyses were utilized to assess the effect of risk scores on OS and MFS.

### 2.5 Explorations of associations immune-related prognostic signature and immune cells infiltration

To explore the associations between prognostic model and immune cells infiltration, we performed single sample gene set enrichment analysis(ssGSEA).Twenty-nine immune marker gene sets were defined according to immune genome function (immune gene set included immune cell type, function, and pathway), and the enrichment level in each synovial sarcoma sample was quantified and ranked through ssGSEA. Furthermore, we conducted a Wilcoxon rank-sum test to compare the differential abundance of immune cells in the two groups which are according to the status of metastasis and risk score.

### 2.6 Immune cell surface markers expression in SS patients

To analyze the possible immune-related mechanisms of metastasis in patients with synovial sarcoma, we firstly analyzed the correlation of immune checkpoint expression in the two groups of patients. We used GSE40021 data and clinical samples from our center to analyze the expression of immune checkpoints in metastatic and nonmetastatic patients, including B and T-lymphocyte-associated protein (BTLA), programmed death receptor-1(PD-1), programmed death ligand-1(PD-L1), cytotoxic T-lymphocyte-associated protein 4(CTLA4), and lymphocyte-activation gene-3 (LAG-3).

The ssGSEA method indicated that CD4+ T lymphocytes and macrophages showed significantly high infiltration in patients with metastasis or patients with high-risk scores, while NK cells showed significantly low infiltration in these patients. Therefore, we used our clinical samples to perform RT-qPCR verification analysis of CD4, CD68, CD206, and CD56.

### 2.7 Induced polarization of M2 type macrophages

M2 type macrophages were obtained as following methods. THP1 cells were pre-treated with 100 ng/mL PMA for 24h to polarized to THP1-Mφ. Then the supernatant was replaced with fresh RPMI medium with 20 ng/mL IL-4 and 20 ng/mL IL-13 and cells was further cultured for 48h to obtain M2 type macrophages.

### 2.8 Effects of M2 type macrophages on SW982 cells

For cell clone formation assay, SW982 cells were inoculated in six-well plates at a density of 1000 per well for 24h and then cultured with M2-CM for 2 weeks. Cells were washed twice with PBS and fixed with 4% paraformaldehyde for 10 min, then stained with 0.1% crystal violet staining solution for 10 minutes. After washed with water, cells were air-dried and photographed, and finally the colony number was counted.

For CCK8 assay, SW982 cells were placed in 96-well plates at 4000 per well for 24h and then treated with M2-CM for 24h. Each well added 10 μL of CCK8 and cells were cultured for 1h. Absorbance at 450 nm was measured with enzyme linked immune-analyzer(Biotek, USA).

For migration assay, SW982 cells were inoculated in upper transwell chamber at a density of 50,000 per chamber, and each well was added with M2-CM in 24-well plates. Then the chambers were placed in the well for 24h. The chambers were then stained with 0.1% crystal violet staining buffer, and the amount of cells was counted.

For wound closure assay, SW982 cells were inoculated in six-well plates at a density of 300,000 per well until 90-95% confluency. A 200-μL pipette tip was used to create wound by scratch on the cell monolayer, and cells were washed twice with PBS. Wound closure was recorded by photographing at 0h, 24h, and 48h, respectively. Scratch area was calculated by Image J software.

### 2.9 Tissue microarray

For SS tissue microarray staining, IHC of anti-CD56, -CD68 and CD206 were performed. SS tissue array was obtained from SYSUCC with informed patient consent and approval by the Ethics Committee. IHC scores were obtained by the multiplicative score method to evaluate protein expression as previously described. This system includes the intensity and the area of positive cell staining. Average intensity scores from 0 to 3 (representing no staining and weak, intermediate, and strong staining, respectively) and percentage scores from 1 to 6 (representing 1%–4%, 5%–19%, 20%–39%, 40%–59%, 60%–79%, and 80%–100%, respectively) were assigned, and then the 2 scores were multiplied to get values from 0 to 18.

### 2.10 Real-time qPCR experiments

RNeasy Mini Kit (Qiagen) was used to extract total cellular RNA and the first strand DNA was synthesized by First Strand cDNA Synthesis Kit (Fermentas), according to the manufacturer’s protocol. Primers are the following (**Table 4**).

**Table 4:** The primers of genes.

Gene	Forward primer	Reverse primer
GREM2	TTTCCCTGTCCTTGTT CCTG	TGCACCAGTCACTCTTGAGG
CTSS	CCATTGGGATCTCTG GAAGAAAA	TCATGCCCACTTGGTAGGTAT
TINAGL1	ACCAGGTCACTCCTG TCTACC	TGCCTCCCTTGTATAGGAAGAA
ACKR1	CCCTCAACTGAGAAC TCAAGTC	AGGTTGGCACCATAGTCTCCA
HLA-DRB	CGGGGTTGGTGAGAG CTTC	AACCACCTGACTTCAATGCTG
STC2	GGGTGTGGCGTGTTT GAATG	TTTCCAGCGTTGTGCAGAAAA
CD4	CCTCCTGCTTTTCATT GGGCTAG	TGAGGACACTGGCAGGTCTTCT
CD68	CGAGCATCATTCTTTC ACCAGCT	ATGAGAGGCAGCAAGATGGACC
CD206	AGCCAACACCAGCTC CTCAAGA	CAAAACGCTCGCGCATTGTCCA
CD56	CATCACCTGGAGGAC TTCTACC	CAGTGTACTGGATGCTCTTCAGG

### 2.11 Data analysis

All analyses were performed using R (version 3.5.1). Unless otherwise noted, P<0.05 was considered significant.

## 3 Results

### 3.1 Identification and characterization of DEIRGs in SS

**Figure 1:**
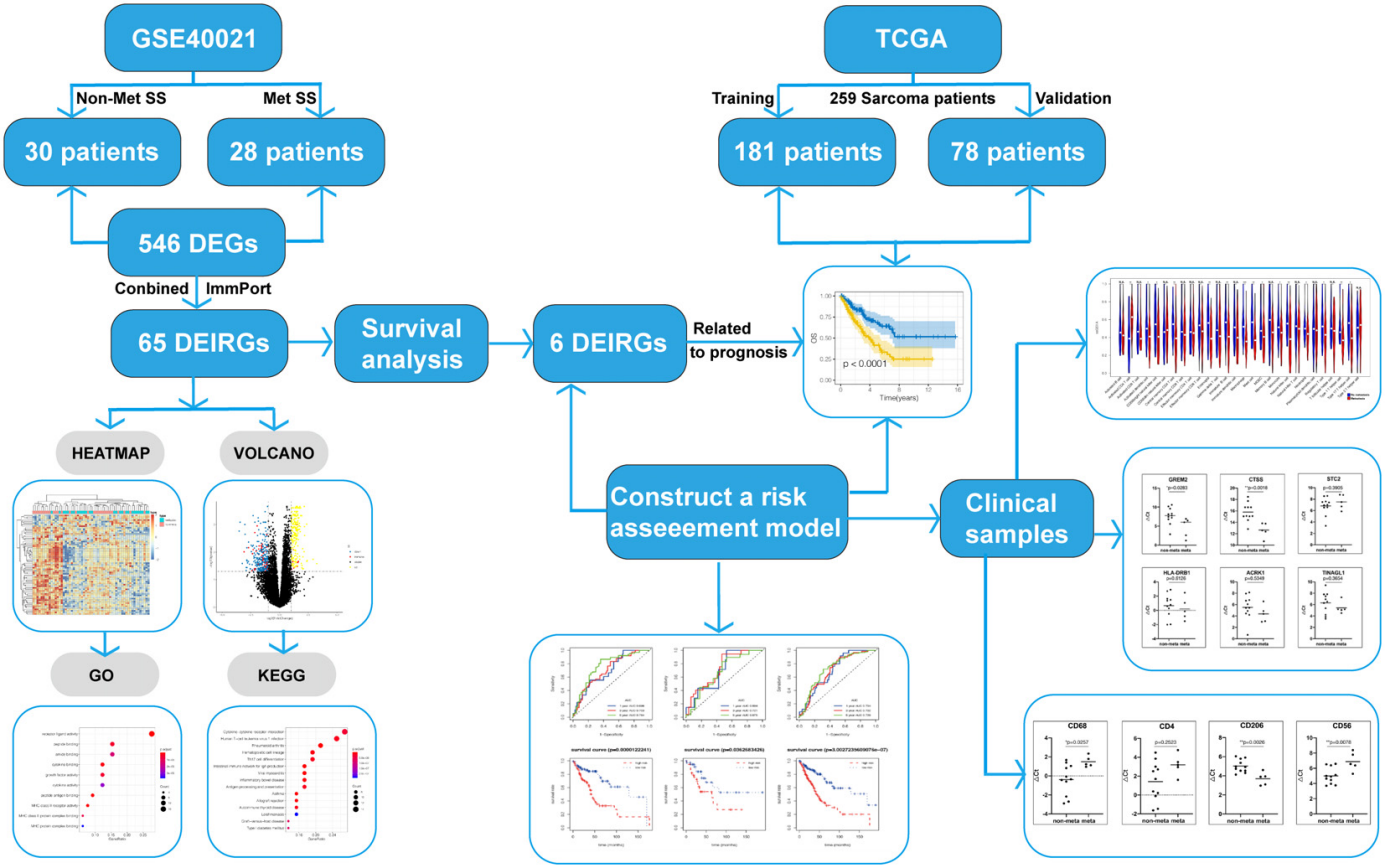
The overall screening flow of chart.

**Figure 2:**
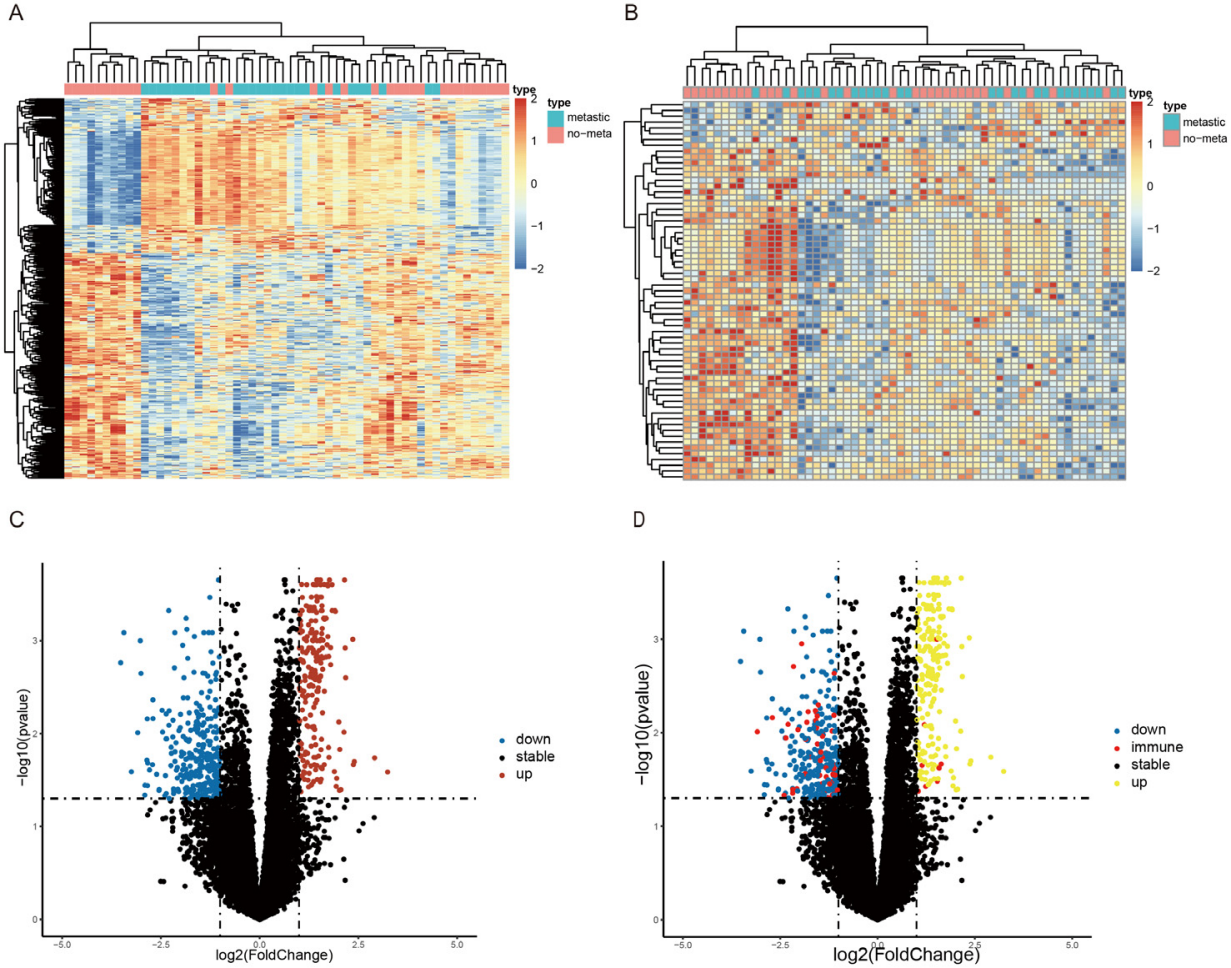
Identification of differentially expressed immune-related genes (DEIRGs) associated with SS metastasis. Heatmap of the top **(A)** 546 DEGs and **(B)** 65 DEIRGs associated with SS metastasis in the GSE40021 datasets. The color from blue to red represents the progression from low expression to high expression. Volcano plot of **(C)** DEGs and **(D)** DEIRGs detected from the GSE40021 datasets. The red dots represent upregulated genes with statistical significance, and the blue dots represent downregulated genes with statistical significance. The red dots represent immune-related genes with statistical significance, and the black dots represent no differentially expressed genes.

A total of 546 DEGs(225 upregulated and 321 downregulated) and 65 DEIRGs (8 upregulated and 57 downregulated) were identified as differentially expressed in metastatic SS compared with nonmetastatic patients. The overall screening flow chart is shown in the flow chart (**Figure 1**). The heat maps revealed that metastatic SS can be obviously distinguished from the nonmetastatic patients according to DEGs and DEIRGs (**Figures 2A, B**). Volcano plots shows the distribution of differentially expressed genes between metastatic SS and nonmetastatic controls (**Figures 2C, D**).

**Figure 3:**
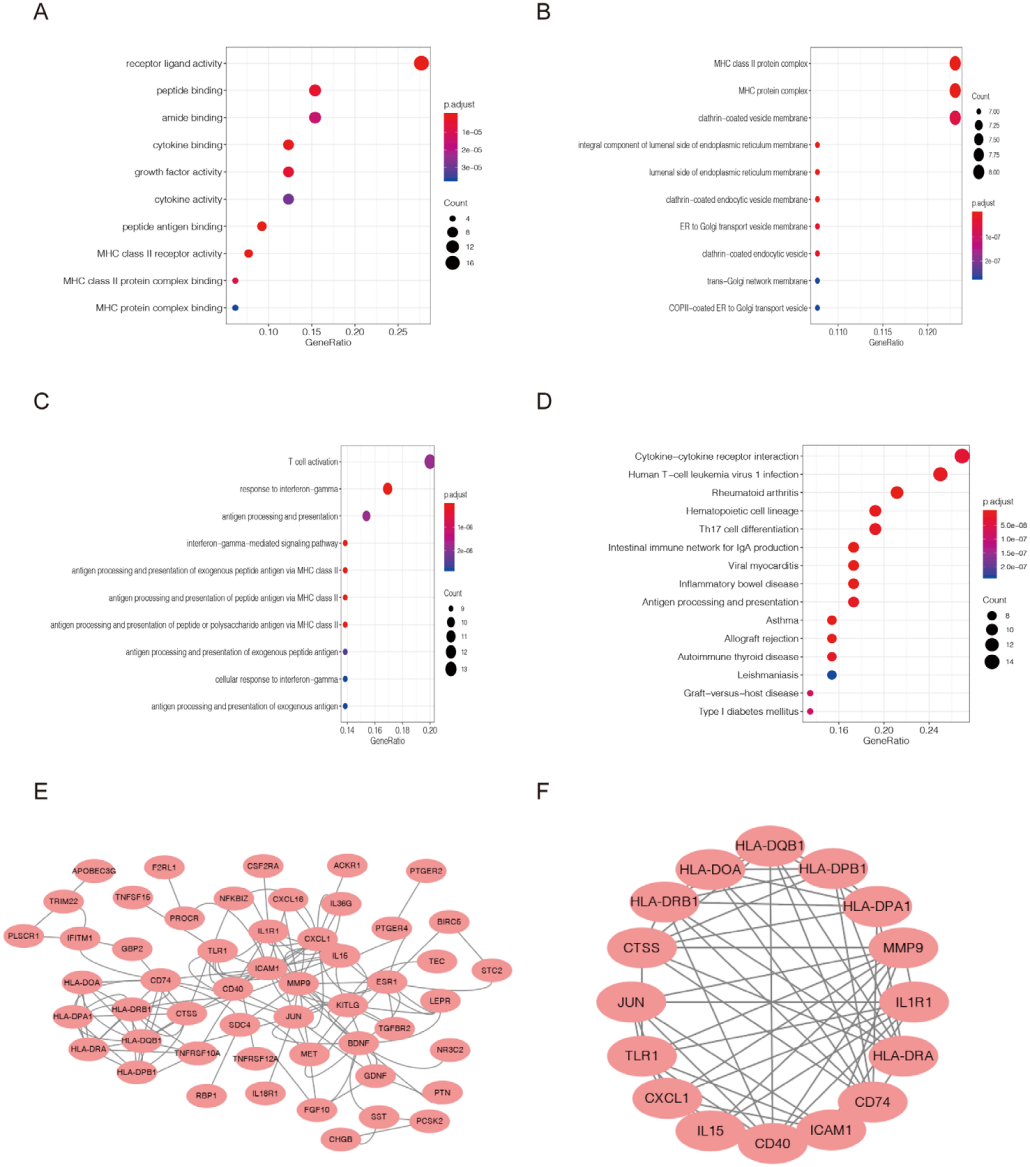
Functional enrichment analysis of differentially expressed immune-related genes(DEIRGs). Gene Ontology analysis representing **(A)** biological process, **(B)** cellular component, and **(C)** molecular function. **(D)** The top 10 most significant Kyoto Encyclopedia of Genes and Genomes (KEGG) pathways. **(E, F)** Protein–protein interactions of DEGs were analyzed using the STRING database. The most significant module in the PPI network was identified using the MCODE plugin.

The 65 DEIRGs were further analyzed by GO and KEGG analysis. GO analysis revealed that primary functional categories in the biological processes (BP) were T cell activation, γ-interferon response, and antigen processing and presentation (**Figure 3A**). For cellular components (CC), the major enriched GO terms were MHC II protein complex, MHC protein complex, and clathrin-coated vesicle membrane (**Figure 3B**). The molecular functions (MF) mainly included the receptors ligands activity, peptide, amino and cytokine binding (**Figure 3C**). KEGG pathway indicated that the DEIRGs were mainly involved in human leukaemia virus-1 infection, rheumatoid arthritis, antigen processing and presentation and other related pathways (**Figure 3D**).

Here, we utilized Cytoscape to construct and visualize the main regulatory network. As shown in **Figure 3E**, protein–protein interactions(PPI) of DEGs were analyzed using the STRING database. The most significant module in the PPI network was identified using the MCODE plugin. This protein regulatory network revealed the regulatory relationships among these immune-related genes (**Figure 3F**).

### 3.2 Construction of the immune-related prognostic model for SS

**Figure 4:**
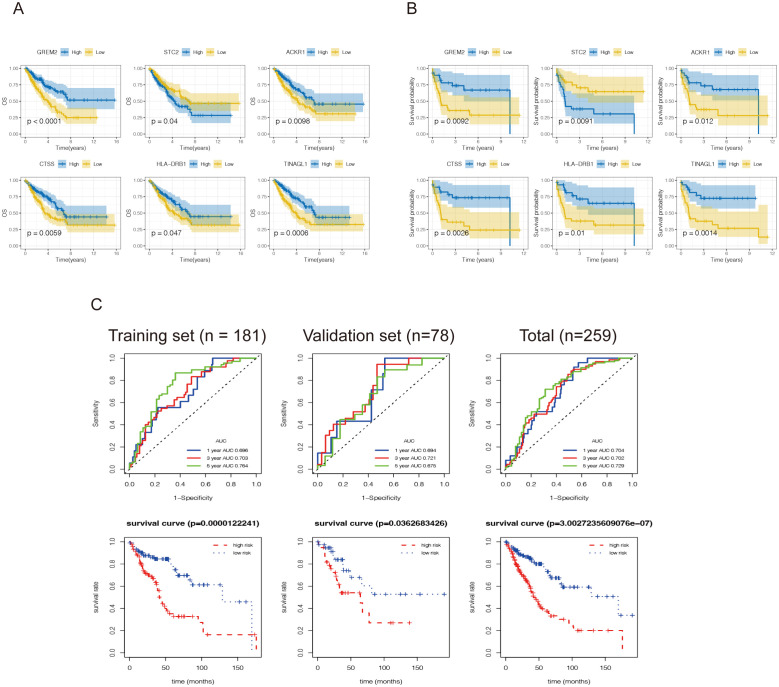
Construction of an immune-related prognostic model for synovial sarcoma. Kaplan-Meier curves for **(A)** overall survival and **(B)** metastasis-free survival analysis of the DEIRGs in SS patients. The blue line represents the high expression group, and the yellow line denotes the low expression group. The figure shows the Kaplan-Meier curves of the six genes screened with significant differences. **(C)** A total of 181 patients were included in the training set, and the remaining 78 patients were included in the validation set. Receiver operating characteristic curve (ROC) analysis predicted overall survival using the risk score. Time-dependent ROC curves of the OS signatures at 1, 3, and 5 years. Survival curves for the low-risk and high-risk groups. The red line represents the high-risk score group, and the blue line denotes the low-risk score group.

65 DEIRGs were subjected to Lasso Cox regression analysis, and 6 genes were filtered out. The results showed that only 5 upregulated genes (GREM2, CTSS, TINAGL1, ACKR1, and HLA-DRB1) and one downregulated gene (STC2) were independently related to prognosis. The OS and MFS were presented in **Figures 4A, B**. Then multivariate Cox analysis were performed and 6 genes were finally selected to establish a prognostic model. The formula was shown as: risk score = (-0.056 * GREM2 expression level) + (-0.285 * CTSS expression level) + (0.297 * STC2 expression level) + (-0.180* TINAGL1 expression level)+(0.003*ACKR1)+(0.052*HLA-DRB1 expression level).All the six genes were risky prognostic genes with hazard ratio > 1. Risk scores were based on genes expression levels multiplied its corresponding regression coefficients. Regression coefficients were calculated by multivariate Cox regression. The risk scores were not only related to the expression levels of these genes, but also related to the correlation coefficients.

Then 259 high grade soft tissue sarcoma(STS) samples in TCGA were utilized again and randomly classified into a training set (n = 181) and validation set (n=78). First, according to the formulas generated in the training set, the risk scores were calculated, including the risk score based on the OS signature. The ROC curves revealed that the discrimination of both signatures in the validation set and total set were favorable, with the AUC ranging from 0.694-0.721,0.702-0.729,respectively (**Figure 4C**), indicating that the prognostic model had good sensitivity and specificity. Subsequently, according to the optimal risk score cutoff identified by X-tile, STS patients were stratified into high-risk and low-risk groups from training set and validation set. The K-M curves indicated that patients in the high-risk group had a worse OS than those in the low-risk group. These results revealed that both signatures were valuable tools for predicting the prognosis of STS patients.

### 3.3 Immune-related prognostic model can predict immune cell infiltration

**Figure 5:**
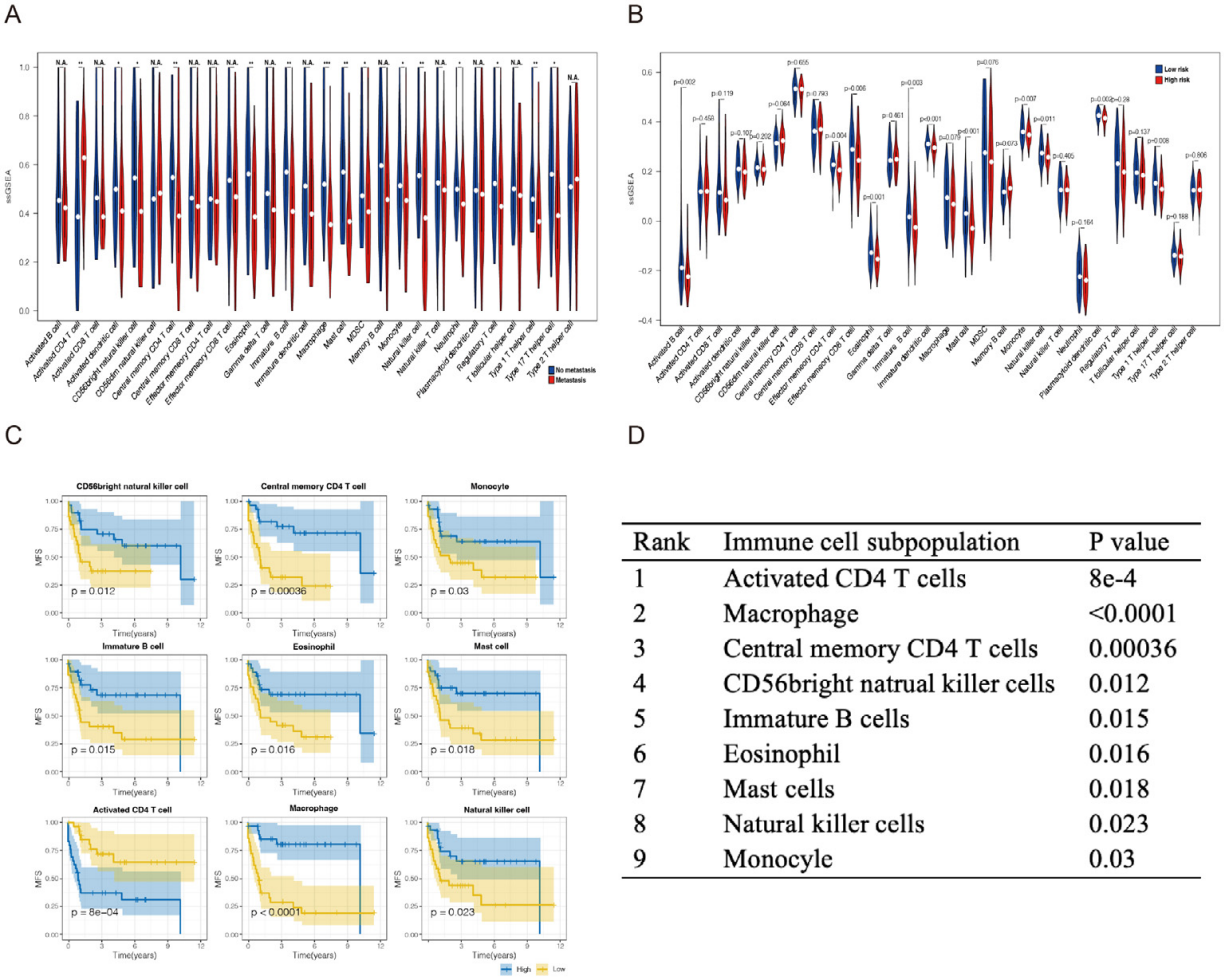
Analysis of the immune landscape of GSE40021 patients. Immune infiltration landscape analyzed by the ssGSEA score−based method in synovial sarcoma from the GSE40021 dataset. **(A)** The blue square represents the no metastasis group, and the red square denotes the metastasis group. **(B)** The blue square represents the high-risk-score group, and the red square denotes the low-risk-score group. **(C)** Infiltrating immune cells were significantly associated with improved prognosis. The high- and low-score groups were divided based on the top 30% and the bottom 30% infiltrating scores calculated by the ssGSEA algorithm, respectively. **(D)** The subpopulations of immune cells associated with prognosis were ranked in descending order based on the magnitude of their differences. *P<0.05; **P<0.01; ***P<0.001. NA, Not Applicable.

Given the important roles of infiltrating immune cells in the tumor microenvironment, we integrated the comprehensive analysis of immune-related prognostic signature combined with immune infiltrates. Utilizing the ssGSEA and CIBERSORT algorithms, we assessed and compared the infiltration abundance of immune cells based on metastatic status and the risk scoring model. The results indicated that the risk scoring model effectively predicted the immune cell infiltration across various metastatic states of SS. (**Figures 5A, B**; **Supplementary Figures S1A, B**). It is further indicating that the Immune-related prognostic model can assess the infiltration of immune cell well for SS.

In addition, we investigated the relationship between six hub immune signatures and several key immune cell subpopulations. Further results revealed that the infiltration of nine immune cell subpopulations correlates with prognosis, indicating that these subpopulations may significantly contribute to metastasis in SS patients. Consequently, we ranked the P-values and selected the top three immune cell subpopulations for subsequent experiments: CD4+T lymphocytes, macrophages, and NK cells (**Figures 5C, D**).

### 3.4 The metastatic mechanism of SS may not be related to the expression of immune checkpoints

**Figure 6:**
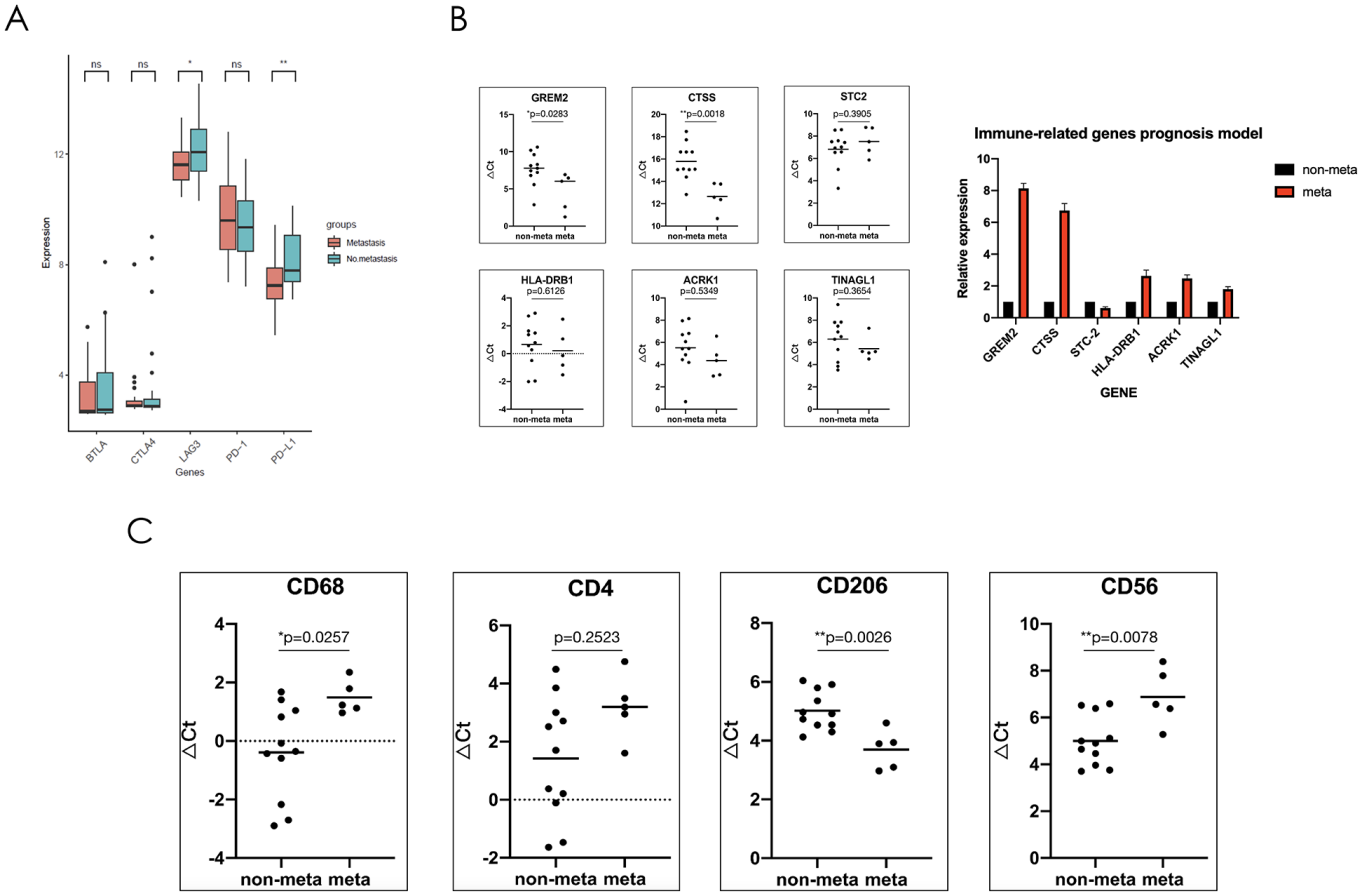
The immune mechanism of metastatic synovial sarcoma may be due to the reduction of NK cell and macrophage infiltration in the tumor microenvironment as well as polarization of macrophages towards M2, forming an immunosuppressive microenvironment. **(A)** Comparison showing the mRNA expression levels of immune checkpoints in the GSE40021 dataset. **(B)** Sixteen synovial sarcoma patient samples were used to verify the mRNA expression levels of six genes, which were used to construct an immune-related risk assessment model. **(C)** mRNA expression levels of immune cell markers. CD68, total macrophage surface marker; CD4, total helper T cell surface marker; CD206, M2-type macrophage surface marker; and CD56, total NK cell surface marker. *P<0.05; **P<0.01; ns, not significant.

To further explore the relevant immune metastatic mechanism of SS, we utilized the GSE40021 dataset to divide patients into two groups (metastatic and nonmetastatic) and analyzed the following immune checkpoints: BTLA4, CTLA4, PD-1, PD-L1, and LAG3. The results suggested that there was no significant difference in the expression of BTLA4, CTLA4 and PD-1. In addition, the expression of PD-L1 and LAG3 was lower in metastatic compared to nonmetastatic patients (**Figure 6A**). Previous clinical trials with subgroup analysis of SS have indicated that the PD-1 inhibitors has poor efficacy. Thus, we believe that the immune metastatic mechanism of SS is not caused by the surveillance pathways of evading immune checkpoints.

### 3.5 Metastatic SS patient show significant expression of the GREM2 and CTSS genes, reduced activated NK cell and macrophage infiltration, and polarized M2 macrophages in the tumor microenvironment

To further verify the results obtained through public database analysis, we collected 16 patients with SS who had follow-up data within three year in our center (Clinical features are shown in **Table 2**). RNA was extracted from tumor tissue, and the expression of DEIRGs including GREM2, CTSS, TINAGL1, ACKR1, HLA-DRB1, and STC2 was verified using real-time quantitative reverse transcription PCR (RT-qPCR). Compared to non-metastatic patients, the GREM2 and CTSS genes exhibited significantly higher expression levels, while TINAGL1, ACKR1, and HLA-DRB1 showed a tendency to increase in metastatic soft tissue sarcoma (SS); however, these differences were not statistically significant. Additionally, the expression of the STC2 gene was found to be decreased in metastatic patients, but again, no significant difference was observed between the two groups (**Figure 6B**).

To further explore the possible immune mechanism of metastatic SS, we performed RT-qPCR experiment to verify the expression of the immune cell surface marker. In preliminary work, we found the differences of immune cell infiltration between the two groups by the ssGSEA algorithm, and the results showed that there were nine types of immune cell were related to prognosis. We further selected which types of greatest difference in immune cell infiltration or survival prognosis for analyzing. Finally, the following three types of cells were screened: activated CD4+ T lymphocytes, macrophages, and NK cells. We then selected their surface marker genes (CD4, CD68, CD206, and CD56) to verify the expression differences between the two groups by RNA levels. The RT-qPCR results indicated that the CD68 and CD56 genes were expressed at low levels in patients with metastatic SS, but the CD206 gene was highly expressed. These results were significantly different between the two groups. The gene expression of the CD4 tended to increase in patients with non-metastatic SS, but there was no significant difference (**Figure 6C**).

### 3.6 M2-type macrophages contribute to the metastatic phenotype of synovial sarcoma cells

**Figure 7:**
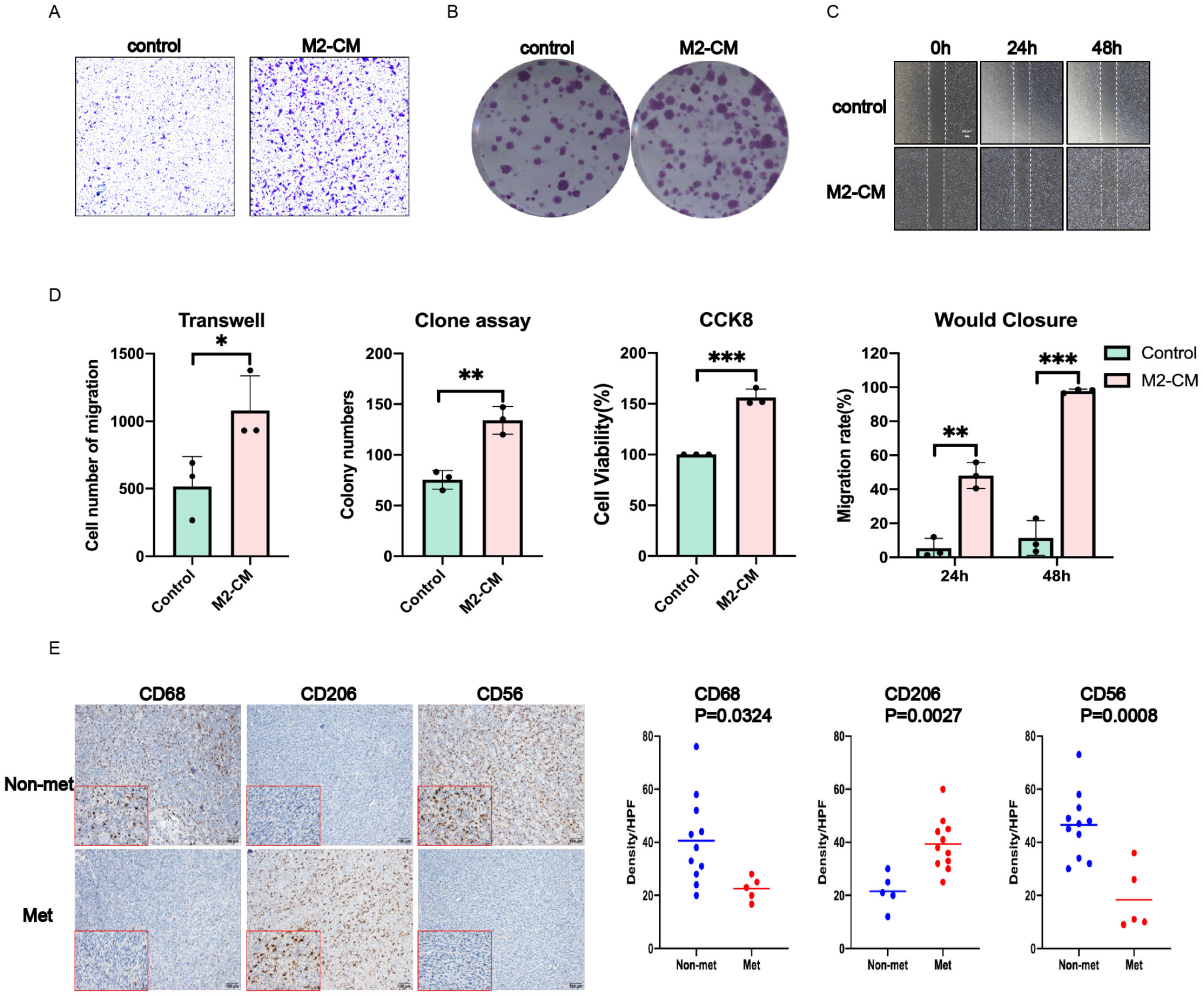
M2-type macrophages contribute to obtain the metastatic phenotype of SW982 cells. The supernatant of M2 type and tumor cells were co-cultured for 48 hours before performing functional experiment *in vitro*. **(A)** Transwell assay. **(B)** Cell clone formation assay. **(C)** Wound closure assay. **(D)** Statistical results of the above and CCK8 experiments. **(E)** Left: Representative images of IHC staining for CD68,CD206 and CD56 in tumors of the SS patients. Right: The density of CD206 TILs was much higher in tumors of the SS patients; The density of CD68 and CD56 was much lower in tumors of the SS patients. Data indicate the mean ± SD. *P < 0.05, **P < 0.01, ***P < 0.001.

In order to further verify whether M2-type macrophages could promote tumor metastasis and colonization, we co-cultured synovial sarcoma SW982 cells with the conditional medium of M2-type macrophages (M2-CM), and set normal medium as control. We first induced the differentiation of mouse monocytes to M2-type macrophages and then collected the prepared supernatant as conditional medium. These results showed that compared with the control group, M2-CM could significantly enhance the proliferation and migration of synovial sarcoma cells, as shown in **Figures 7A–D**.

In view of the favorable results *in vitro*, we carried out immunohistochemical verification of tissue samples from SS patients, and mainly observed the infiltration of M2-type macrophages and NK cells. Compared with the control group, the results showed significantly higher expression of CD68, CD206 and lower expression of CD56 in tumors of metastatic SS patients, which indicated more infiltration of M2 macrophages, while the infiltration of NK cells was significantly reduced, as shown in **Figure 7E**.

## 4 Discussion

Large-scale omics analyses, encompassing mRNA, microRNA (miRNA), protein, DNA sequence, methylation, and copy number alterations, have revealed that distinct molecular subtypes of soft tissue sarcomas exhibit genomic and regulatory diversity in their driver pathways (13, 14). Moreover, the immune microenvironment, as inferred from DNA methylation and mRNA profiles, correlates with patient outcomes and may offer valuable insights for clinical trials involving immune checkpoint inhibitors. Although synovial sarcoma is characterized by a simple karyotype and a translocation involving SS18 and SSX1, 2, or 4, omics studies are particularly crucial for patients who share the same karyotype but exhibit differing clinical prognoses (15, 16). Our study revealed significant differences in the immune microenvironment between the two patient groups, thereby corroborating the conclusions of the aforementioned article to some extent. Therefore, it is important to clarify the relevant molecular metastatic mechanisms of SS and to identify new immune-related therapeutic targets and prognostic markers.

Previous studies have demonstrated that the infiltration of various lymphocyte subpopulations within the tumor microenvironment is closely associated with tumor recurrence and metastasis (17–19). The T-cell receptor therapy can be used to effectively target soft tissue sarcoma and provides rationale to expand this approach to other solid malignancies. Changes in the tumor immune microenvironment are crucial to the malignant progression of soft tissue sarcoma. Consequently, the development of immune-related prognostic models holds significant importance for the clinical management of patients with synovial sarcoma (20–23). At present, screening prognostic-related genes and constructing predictive models through multiomics sequencing have shown great predictive potential in a variety of tumor types (24, 25). To the best of our knowledge, it lacks risk assessment model for immune-related genes in patients with metastatic SS. The present study aimed to provide clinicians with accurate and important references for the prognosis of SS patients. To characterize the infiltration of immune cells in the tumor microenvironment, we further explored the relationship between immune-related prognostic models and immune cell infiltration. We found that activated B lymphocytes, effector memory CD4+ lymphocytes, effector memory CD8+ T lymphocytes, eosinophils, mast cells, monocytes, NK cells, plasma-like DC cells, and TH1 helper T lymphocytes had significantly low infiltration in patients with high-risk scores. Among them, eosinophils, monocytes, mast cells, and NK cells showed a significant difference in the metastasis-free survival time of low-risk patients, further suggesting that the immune-related prognostic model has a good ability to assess the infiltration of certain immune cells. At the same time, we found that there was a significantly low infiltration of macrophages and CD4+ T lymphocytes in patients with metastatic synovial sarcoma in the GSE40021 dataset. At present, there are few studies on mast cells and eosinophils in tumor metastasis. It remains unclear whether mast cells and eosinophils are involved in the regulation of the immune microenvironment of synovial sarcoma. Therefore, we focused on NK cells, macrophages, and CD4+ T lymphocytes.

Sutherland et al. found that the decrease in infiltration of NK cells rather than cytotoxic CD8+T lymphocytes in the tumor microenvironment is the main reason for the metastasis and spread of primary tumors by conducting a transcriptome analysis of the primary tumors of small cell lung cancer patients and transgenic mice (26). Ren et al. found that the metastasis regulation of tumor-infiltrating neutrophils is mediated by the activation state of NK cells in a mouse model of breast cancer (27). The infiltration of NK cells in tumors plays a crucial role in regulating tumor metastasis. Kuo et al. cocultured tumor cells with macrophages and collected the supernatant for proteomic analysis, and they showed that the tumor cells secrete succinate into the microenvironment and are activated by binding to succinate receptors on macrophages. Polarization to M2-type macrophages ultimately promotes tumor invasion and metastasis (28). Ruffell et al. reported that macrophages are important participants in the regulation of tumor microenvironment homeostasis. Tumor cells promote the polarization of macrophages to the M2-type by secreting different mediators, which may be driven by local hypoxia and fibrosis of the tumor microenvironment. The increased infiltration of these M2-type macrophages leads to tumor recurrence and metastasis caused by inhibiting the recruitment of cytotoxic T lymphocytes and regulating other aspects of tumor immunity (29). Reviewing our study, we cultured tumor cells with M2-CM and found that SS cells transformed into a more aggressive phenotype which manifested enhanced invasive and colonizing abilities. Further verification of clinical samples showed that more M2-type macrophages were infiltrated in tumors of metastatic SS patients, suggesting that the metastatic mechanism may be accomplished through interaction with macrophages in the tumor microenvironment, and lead its polarization towards M2 type, which promoted tumor cells to acquire an invasive phenotype for further tumor progression.

A recent review published by Lam et al. proposed that CD4+ T lymphocytes have a variety of different cell subpopulations. The balance between immunosuppressive Tregs and the proinflammatory TH17 cell subpopulation plays an important regulatory role in the occurrence and metastasis of lung cancer. Several studies have shown that these two CD4+ T cell subgroups play an active role in promoting lung cancer progression and metastasis (30). The infiltration of NK cells, eosinophils, macrophages, and CD4+ T lymphocytes in tumors plays a crucial role in regulating tumor metastasis. To further verify the role of these three immune cell subpopulations in patients with metastatic synovial sarcoma, we enrolled synovial sarcoma patients with three years follow-up data in our center. In patients with metastatic SS, the infiltration of macrophages was significantly increased, and the infiltration of NK cells was significantly reduced. Furthermore, we also found that the infiltration of M2-type macrophages was the main infiltration, while the infiltration of CD4+ T lymphocytes was not significantly different. Our analysis of the GEO database revealed that most immune checkpoints did not exhibit significant differences between metastatic and nonmetastatic synovial sarcoma (SS) patients. Furthermore, we observed low expression levels of LAG3 and PD-L1 in metastatic patients. These findings indicate that the metastasis of synovial sarcoma is unlikely to be driven by the evasion of immune checkpoint surveillance. The results of previous study analyzing genetic alterations across various sarcoma types through targeted sequencing to identify effective targeted agents revealed that the most prevalent targetable changes involved cell cycle control, TP53, receptor tyrosine kinase/PI3K/RAS pathways, and epigenetic regulators (31). Epigenetic alterations can facilitate cancer progression by influencing the activation, differentiation, and function of immune cells, including T cells and NK cells. Consequently, epigenetic agents may provide therapeutic benefits for patients with metastatic synovial sarcoma (32, 33). Another studies have indicated that synovial sarcoma is classified as a cold tumor, with the behavior of malignant tumor cells being regulated by the SS18-SSX fusion protein. This regulation is inhibited by cytokines secreted by macrophages and T cells, a finding that aligns with our own results. Importantly, the characteristics of these macrophages appear to predominantly reflect the M2 subtype, suggesting that the malignant features associated with their metastasis may be closely linked to the infiltration of M2 macrophages and their cytokine secretion. Based on these data, it is plausible to speculate that the immune mechanism underlying metastasis may involve tumor cells inhibiting the secretion of specific cytokines, leading to a reduction in the infiltration of natural killer (NK) cells and macrophages within the tumor microenvironment. Additionally, we found that the infiltrating macrophages predominantly polarized to the M2 phenotype, which contributed to the formation of an immunosuppressive tumor microenvironment, thereby facilitating lung metastasis.

The incidence of SS is approximately 1 in 100,000 globally, and there is a limited number of studies addressing this condition. In contrast to more prevalent tumors, such as lung cancer, even minor findings represent significant advancements in the diagnosis and treatment of SS. To our knowledge, few researchers have developed a prognostic model based on immune-related genes from an immunotherapeutic perspective; therefore, the development of this model holds considerable clinical significance and represents a pioneering effort in the field. Current clinical trials have identified that certain patients with SS respond positively to immunotherapy, making the identification of these patients crucial for effective treatment (34, 35). Our results also found a significant increase in the infiltration of M2-type macrophages in tumors of metastatic SS, which were validated *in vitro* at RNA and protein levels, respectively. We also co-cultured the conditioned medium of M2-type macrophages with tumor cells and observed that tumor cells acquired a more invasive phenotype *in vitro*. This finding suggests that M2-type macrophages may play a significant role in promoting tumor metastasis. Understanding the molecular mechanisms underlying their interaction could reveal new immune escape mechanisms and identify relevant therapeutic targets. Current studies indicate that tumor cells can evade macrophage phagocytosis by binding to the SIRPα receptor on macrophages through the overexpression of CD47. This area of research holds considerable promise for advancing our understanding of tumor immunity (36, 37). Monotherapy and combination therapies targeting the CD47-SIRPα axis are currently undergoing clinical trials. To our knowledge, our current findings have yet to be substantiated by high-level evidence in studies concerning SS, thus emphasizing the importance of our ongoing in-depth investigations.

Our study also had several limitations. Firstly, the survival analyses of the identified DEIRGs were validated for all sarcomas and not specifically synovial sarcoma in TCGA because there is currently no database of large samples of synovial sarcoma patients with follow-up information. Existing evidence shows that immune-related prognostic models can predict SS metastasis and the infiltration of immune cell in the tumor microenvironment. Due to the low incidence of SS, there is a certain objective difficulty in collecting cases. Consider the rare morbidity of SS, although the number of cases is not large, it is still sufficient of statistical or methodological performance to be used as the basis for the study. Future research will require a large number of clinical samples and multiomics analysis.

## 5 Conclusion

In conclusion, for the first time, several immune-related genes were detected to be significantly related to SS prognosis by comprehensive analyses. Moreover, we constructed a novel immune-related prognostic model as an independent prognostic predictor for SS. This prognostic model may also serve as predictor for the immune cells infiltration, proving its key role in tumor immune microenvironment. This may explain the poor efficacy of PD-1 inhibitors in synovial sarcoma. The mechanism of metastasis may be related to the polarization of macrophages towards the M2 type and remodeling of the immune microenvironment. Intervening in advance and reversing the changes in the immune microenvironment to increase the infiltration of NK cells is expected to delay metastasis and improve the survival of patients.

## Data Availability

The datasets presented in this study can be found in online repositories. The names of the repository/repositories and accession number(s) can be found in the article/**Supplementary Material**.

## Supplementary material

Supplementary Figure 1 Analysis of the immune landscape of GSE40021 patients. Immune infiltration landscape analyzed by the CIBERSORT algorithm in SS from the GSE40021 dataset. **(A)** The green square represents the no metastasis group, and the red square denotes the metastasis group. **(B)** The blue square represents the high-risk-score group, and the red square denotes the low-risk-score group.

## Glossary

SS: Synovial sarcoma
TCGA: The Cancer Genome Atlas
IRGs: Immune-related genes
DEGs: Differentially expressed genes
DEIRGs: Differentially expressed immune-related genes
RT-qPCR: Real-time quantitative reverse transcription
BTLA: B and T-lymphocyte-associated protein
PD-L1: programmed death receptor-1
PD-1: programmed death ligand-1
CTLA4: Cytotoxic T-lymphocyte-associated protein 4
LAG-3: Lymphocyte-activation gene-3
GO: Gene ontology database
CC: cellular component
BP: biological process
MF: molecular function
KEGG: The Kyoto encyclopedia of genes and genomes
ROC: Receiver operating characteristic
AUC: Area under the ROC curve
OS: Overall survival. MFS, Metastasis-free survival analysis
LMS: Leiomyosarcoma
DLPS: Dedifferentiated liposarcoma
UPS: undifferentiated pleomorphic sarcoma
MFS: Myxofibrosarcoma
MPNST: Malignant Peripheral Nerve Sheath Tumor
