# Supplementary figures and images for: Development and validation of a novel immune-related prognostic model and the potential metastatic mechanism in synovial sarcoma
Source: Front Immunol. 2024 Dec 10;15:1448464. doi: 10.3389/fimmu.2024.1448464 (PMC11671775; doi:10.3389/fimmu.2024.1448464)

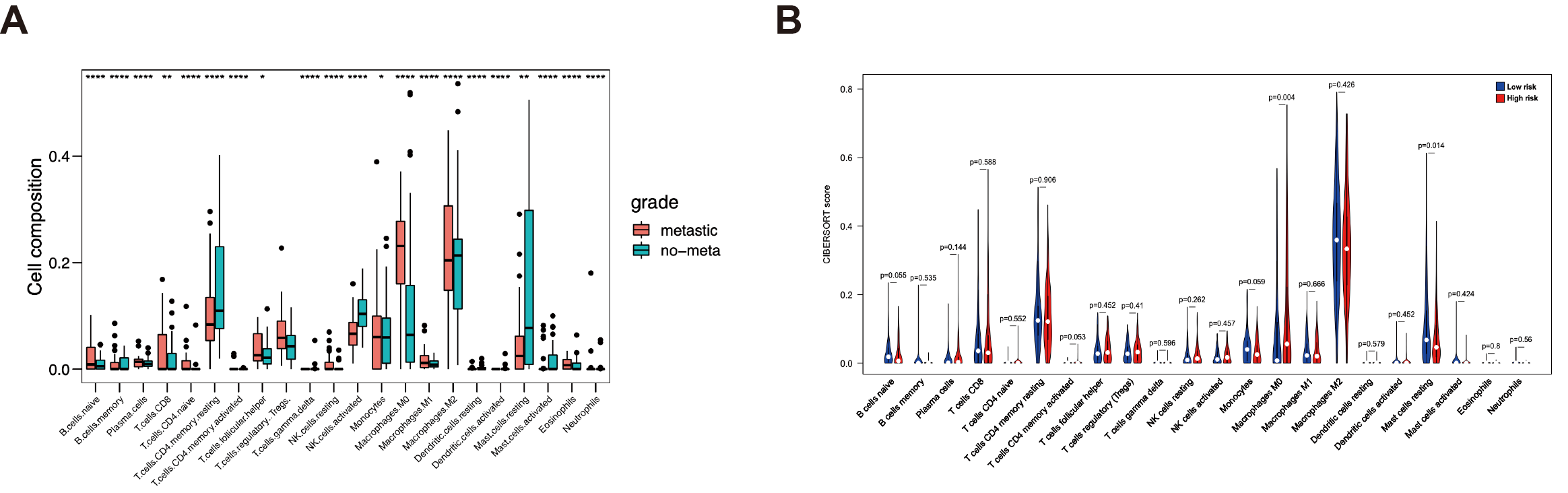

Supplement: Supplementary Figure 1 — Analysis of the immune landscape of GSE40021 patients. Immune infiltration landscape analyzed by the CIBERSORT algorithm in SS from the GSE40021 dataset. (A) The green square represents the no metastasis group, and the red square denotes the metastasis group. (B) The blue square represents the high-risk-score group, and the red square denotes the low-risk-score group. [file Image1.tif]
